# Better health outcomes at lower costs: the benefits of primary care utilisation for chronic disease management in remote Indigenous communities in Australia’s Northern Territory

**DOI:** 10.1186/1472-6963-14-463

**Published:** 2014-10-04

**Authors:** Yuejen Zhao, Susan L Thomas, Steven L Guthridge, John Wakerman

**Affiliations:** Department of Health, Health Gains Planning, Darwin, Australia; Centre of Research Excellence in Rural and Remote Primary Health Care, Bendigo, Australia; Centre for Remote Health, Flinders University and Charles Darwin University, Alice Springs, Australia; Flinders Northern Territory, Darwin, Australia

**Keywords:** Indigenous, Northern Territory, Australia, Remote, Chronic disease, Primary care, Cost effectiveness, Hospitalisation, Mortality, YLL

## Abstract

**Background:**

Indigenous residents living in remote communities in Australia’s Northern Territory experience higher rates of preventable chronic disease and have poorer access to appropriate health services compared to other Australians. This study compared health outcomes and costs at different levels of primary care utilisation to determine if primary care represents an efficient use of resources for Indigenous patients with common chronic diseases namely hypertension, diabetes, ischaemic heart disease, chronic obstructive pulmonary disease and renal disease.

**Methods:**

This was an historical cohort study involving a total of 14,184 Indigenous residents, aged 15 years and over, who lived in remote communities and used a remote clinic or public hospital from 2002 to 2011. Individual level demographic and clinical data were drawn from primary care and hospital care information systems using a unique patient identifier. A propensity score was used to improve comparability between high, medium and low primary care utilisation groups. Incremental cost-effectiveness ratios and acceptability curves were used to analyse four health outcome measures: total and, avoidable hospital admissions, deaths and years of life lost.

**Results:**

Compared to the low utilisation group, medium and high levels of primary care utilisation were associated with decreases in total and avoidable hospitalisations, deaths and years of life lost. Higher levels of primary care utilisation for renal disease reduced avoidable hospitalisations by 82-85%, deaths 72-75%, and years of life lost 78-81%. For patients with ischaemic heart disease, the reduction in avoidable hospitalisations was 63-78%, deaths 63-66% and years of life lost 69-73%. In terms of cost-effectiveness, primary care for renal disease and diabetes ranked as more cost-effective, followed by hypertension and ischaemic heart disease. Primary care for chronic obstructive pulmonary disease was the least cost-effective of the five conditions.

**Conclusion:**

Primary care in remote Indigenous communities was shown to be associated with cost-savings to public hospitals and health benefits to individual patients. Investing $1 in primary care in remote Indigenous communities could save $3.95-$11.75 in hospital costs, in addition to health benefits for individual patients. These findings may have wider applicability in strengthening primary care in the face of high chronic disease prevalence globally.

## Background

Australia has made great progress in population health [1]. From 2001 to 2011, life expectancy at birth increased by 2.7 years for Australian males and 1.8 years for females [2]. Unfortunately, life expectancy for the Australian Aboriginal and Torres Strait Islander population (hereafter referred to as Indigenous) has lagged behind, in recent estimates, by 10.6 and 9.5 years for males and females respectively [3]. In the Northern Territory (NT), the life expectancy gap for the Indigenous population is 40-50% wider than the national figures [4]. It has been estimated that chronic diseases contribute 80% of the gap in life expectancy between Indigenous and non-Indigenous NT populations [5]. Preventable chronic diseases (PCDs), including diabetes, ischaemic heart disease (IHD), renal disease, chronic obstructive pulmonary disease (COPD) and hypertension are common in remote Indigenous communities in the NT [6, 7].

These diseases are associated with the social determinants of health, including poorer access to appropriate health services [8]. Indigenous people experience higher rates and earlier onset of PCD and many patients are diagnosed with multiple morbidities [9], adding to the disability and mortality burden [10]. In the NT, 30% of residents of remote Indigenous communities aged over 50 years have at least three chronic conditions [11]. PCDs are often characterised by gradual onset, progression over a person’s lifetime and functional impairment requiring prolonged assistance [12]. PCDs are costly to manage with chronic conditions accounting for 60% of the total health and residential care expenditure in Australia in 2012-13 [13].

In 2008, the Australian Government agreed to targets to address Indigenous disadvantage, including closing the gap in life expectancy within a generation (by 2031) [14]. Access to culturally appropriate, comprehensive primary care was acknowledged as essential in achieving this goal [15]. In the words of the chair of the Close the Gap steering committee for Indigenous health equality: ‘Aboriginal peoples and Torres Strait Islanders have less access to essential health services than other Australians. Too often they don’t get the health care they need, when and where they need it’ [16]. This is often the case in very small remote communities in the NT where there are few local services and residents must rely either on periodic visiting services or travel to access services in larger centres. Primary care is effective in the prevention, early detection and management of chronic disease, and in preventing potentially avoidable hospitalisations (PAH) and associated morbidity and mortality [17]. Evidence has shown a strong association between primary care and better health outcomes, lower costs and greater equity (reduced disparities) in health across population subgroups [17–20]. There is some evidence for strengthened primary care improving health outcomes and cost effectiveness in remote communities in Australia [21–25]. However, a more comprehensive evidence base is needed to support greater investments in primary care for Indigenous people living in remote parts of Australia.

The importance of primary care in remote Indigenous communities has been recognised and there have been increases in expenditure in recent years [26, 27]. However, there have also been increases in the population, rates of PCD and in costs associated with providing primary care, which together have offset anticipated benefits [11]. In communities where primary care services are available, funding levels are often insufficient to meet local health needs [28]. Preventable admissions to hospital remain high relative to less remote regions. Residents of remote NT communities experience poorer access to Medicare and the Pharmaceutical Benefits Scheme (PBS), the Australian Government funded universal health care insurance systems. In the 2003/4 financial year, the per capita Medicare benefit was estimated at $108 for an Indigenous person in a remote community in the NT, compared to $413 for other Australians. After inclusion of additional Indigenous specific primary care funding sources in the NT, including Aboriginal specific medical services and pharmaceutical allowances, the per capita expenditure gap persists [29]. Compared to the national average Medicare and PBS payments, there was an annual primary care funding gap of $102 million in 2012. In 2011, with additional Indigenous specific primary care funding the gap was estimated at $50 million [30]. Sustained investment, proportionate to need, is required to achieve equitable access to primary care for Indigenous Australians living in remote NT communities [11].

The NT covers a large area of Australia, 17% of the country’s landmass, but contains just 1% of the total population [31]. While this makes provision of primary care services more costly compared to metropolitan areas, the costs of acute care are also substantially higher [32, 33]. Public hospitals provide most of the acute care services to remote Indigenous residents [33]. Economic evaluation, including cost-effectiveness studies, can provide important evidence which can be used to maximise health benefits through effective use of scarce health resources [34]. The need for evidence has international significance in relation to strengthening primary care in the face of a global chronic disease epidemic. The aim of this study was to compare the cost-effectiveness of different levels of primary care utilisation for five chronic diseases, measured by recurrent costs, total admissions and PAHs, deaths and years of life lost (YLL).

## Methods

A cost effectiveness analysis was applied to an historical cohort to compare marginal changes in health outcomes and costs between alternative primary care utilisation groups [35, 36]. The study cohort included all Indigenous residents from geographically defined NT remote localities, who were aged 15 years or over on 1 January 2002 and who attended any of the NT Department of Health’s 54 remote clinics or five public hospitals between 1 January 2002 and 31 December 2011.

Three electronic data sources were used: the primary care information system (PCIS), hospital admitted patient data (Caresys), and the government accounting system (GAS). A unique client identifier linked individual records between PCIS and Caresys data. The four health outcome measures analysed were total hospitalisations, PAHs, deaths and YLLs [33]. Deaths were identified using the date of death recorded in PCIS and mode of discharge from Caresys. YLLs were derived by age at death and standard life expectancy used in the second Australian burden of disease and injury study [37].

The study cohort was divided into three groups according to the annual average number of clinic visits: low level primary care (<2 visits annually), medium level primary care [2–11] and high level primary care (≥12). A chi-square test was used to check the baseline comparability between the control and intervention groups in terms of key demographic characteristics and number of chronic diseases. Groups were followed up for hospitalisations and deaths.

Hospitalisations and clinic visits related to the five PCDs were identified using the Australian Refined Diagnosis Related Groups, Version 4 (AR-DRG) [38] and the International Classification of Primary Care, Second Edition (ICPC) (see Table 1) [39]. The International Statistical Classification of Diseases and Related Health Problems, Tenth Revision (ICD-10) was used to identify PAH [40]. PAHs are considered to be admissions that may have been avoided by providing appropriate primary care [40]. The data were stratified by high, medium and low primary care utilisation groups, and analysed separately for the five PCDs (Table 1). To minimise data requirements and avoid complex modelling of multi-morbidities, each disease was analysed independently.

**Table 1:** **List of disease groups and definitions**

Disease group	Primary care ICPC codes	Hospital AR-DRG codes	Hospital ICD-10 codes
Diabetes	T87, T88, T89, T90	F11A, F11B, F13Z, K01Z, K60A, K60B	E10-E14
Ischaemic heart disease	K74, K75, K76	F08A, F08B, F14A, F14B, F14C, F12Z, F01A, F01B, F02Z, F66A, F66B, F74Z, F72A, F72B, F05A, F05B, F06A, F06B, F17Z, F18Z	I20-I25
COPD	R91, R95	E65A, E65B, E69A, E69B, E69C	J40-J44, J47
Renal disease	U88, U90, U95	L65A, L65B, L67A, L67B, L67C, A09A, A09B, L02A, L02B, L60A, L60B, L60C, L61Z	N00-N16
Hypertension	F83, K85, K86	F67A, F67B	I10-I13

Notes: AR-DRG = Australian Refined Diagnosis Related Groups; COPD = Chronic obstructive pulmonary disease; ICD = International Statistical Classification of Diseases and Related Health Problems; ICPC = International Classification of Primary Care.

Both primary care and hospital care were provided free of charge to public patients. The size and complexity of primary care services varied with the population of remote communities, but in 2003/4 an “average” remote clinic was situated 275 kilometres (km) from the nearest hospital (between 100 and 700 km), staffed by 3.4 full time equivalent (FTE) nurses, 1.3 FTE Aboriginal health workers, 1 FTE receptionist and 0.2 FTE aide (physical worker). A District Medical Officer (DMO), located at the nearest major centre, visited these clinics, on average, 35 times per year. Specialist outreach services were provided from the hospitals [32]. Locality codes were used to define the catchment areas of the clinics [41]. If there were multiple residential locality codes for one person, then the residential locality was determined by the highest frequency of recorded visits or hospitalisations.

The costing data were sourced from GAS. The cost estimation for each hospital separation was based on the NT average AR-DRG cost, which used the bottom-up method to apportion expenditure based on individual items of acute care consumption. The cost components included nursing, medical, allied health, non-clinical salaries, pathology, pharmacy, imaging, supply, emergency, theatre, critical care, prostheses, hotel services, and other on-costs. The patient costing model provided data to the National Hospital Cost Data Collection based on nationally agreed methods for allocating costs [42]. Corporate overheads, patient travel and major capital costs were excluded in order to focus on differences in inpatient treatment costs. The average cost for clinic visits was estimated using a top-down method by dividing total remote health care expenditure by the volume of clinic visits from 2008/9 to 2010/11 [32]. This average cost estimate was then applied to each patient in the cohort as a component of the total costs. Incremental total and average costs per hospitalisation or YLL averted were estimated by comparing the medium and high utilisation groups with the low utilisation group. DMOs’ salary and travel expenses were included in the costing. Capital expenditure was excluded from the analysis. The average hospital and clinic visit costs were both converted to the 2006/7 costs for comparison by assuming 5% annual health inflation.

Propensity score matching was performed to improve comparability between primary care utilisation groups in this observational study [43]. Using logistic regression to calculate propensity scores enabled us to correct sample selection bias for key confounders of age, sex and number of chronic diseases. In the absence of more reliable data, the number of chronic diseases served as a proxy measure for the severity of PCDs. Cost-effectiveness was measured by the ratio between annual incremental mean cost of medium and high primary care utilisation groups and the annual incremental mean decrease in those shortfall health outcomes for those groups relative to the low primary care utilisation group [44]. A cost-effectiveness threshold was used as a perceived value when evaluating cost-effectiveness of medium and high primary care utilisation [33]. The standard threshold values were $2915 per separation (NT average cost per AR-DRG) and $120,000 per statistical life year [45]. The net benefits were calculated by the decrease in shortfall health outcomes multiplied by the standard threshold values less costs for medium and high primary care utilisation [44]. The incremental cost-effectiveness ratio was calculated as the incremental cost in primary care per unit of shortfall health outcome averted in comparison to the low utilisation group, under the assumption that the high, medium and low utilisation groups were comparable and the benefit for continued investment was sustainable. The reciprocal of the incremental cost-effectiveness ratio was used as the return-on-investment ratio, indicating the expected marginal benefit per dollar spent on primary care. The average return-on-investment ratio was weighted by the number of patients for each PCD. To assess the uncertainty relating to the cost-effectiveness of primary care, we calculated cost-effectiveness acceptability curves [44], which estimate the probability that primary care is cost-effective in reducing hospital admissions or YLLs, alongside different values placed on a hospital separation or a year of life. The effects and benefits were not discounted.

The NT Department of Health and Menzies School of Health Research Ethics Committee granted research ethics approval for this project (Approval Number: HREC-2012-1849).

## Results

A total of 14,184 remote Indigenous patients were eligible to be included in the study, with a majority (70%) of the cohort aged < 40 years and a minority (44%) were males. Thirty six percent of the cohort had no primary care visits and 15% had no hospitalisations. Of the total number of primary care visits (584,628), 15% were classified as metabolic, 14% circulatory and 13% urological. Of the total number of hospitalisations (137,395), 55% were same-day separations and the average length of stay was three days.

Within the cohort, 49% were in the low primary care utilisation group, 42% were in the medium primary care group and 9% were in the high primary care group. There were no statistically significant differences in age, sex and number of PCDs between the groups after the propensity score matching (*p* >0.05, Table 2). The average costs were $175 per primary care visit and $2915 per hospitalisation in 2006/7 prices.

**Table 2:** **Comparability in age, sex and number of chronic diseases by groups, before and after propensity score matching**

Proportions	Low primary care (n = 6987)		Medium primary care (n = 5926)		High primary care (n = 1271)		χ ^2^significance	
	Before PSM	After PSM	Before PSM	After PSM	Before PSM	After PSM	Before PSM	After PSM
Age								
15-	48%	20%	47%	19%	20%	20%	523.3**	2.04 ^-^
30-	24%	23%	25%	25%	23%	23%		
40-	14%	26%	15%	27%	27%	27%		
50-	7%	18%	8%	17%	17%	17%		
60-	7%	13%	5%	12%	13%	13%		
Sex								
Male	50%	35%	39%	35%	33%	33%	523.3**	2.07 ^-^
Female	50%	65%	61%	65%	67%	67%		
Number of chronic diseases								
0	63%	10%	43%	10%	10%	10%	2004.8**	11.12 ^-^
1	17%	16%	22%	16%	16%	16%		
2	9%	22%	17%	23%	23%	23%		
3	7%	28%	13%	30%	31%	31%		
4	4%	20%	5%	17%	16%	16%		
5	1%	4%	1%	5%	5%	5%		

Note: n = number of patients; - p > 0.05; *p < 0.05; **p < 0.01; PSM = propensity score matching.

Compared to the low utilisation group, the average annual number of hospitalisations per person decreased with increasing levels of primary care for all five PCDs (Table 3). Hospitalisations were reduced by 84% in the medium primary care group and 86% in the high primary care group for renal disease, 78% and 80% respectively for diabetes and 73%-78% for hypertension. The reductions in hospitalisations for IHD and COPD were the lowest among the five PCDs, but still statistically significant, ranging from 61% to 75%.

**Table 3:** **Annual average hospitalisations, avoidable hospitalisation, death rate and years of life lost by primary health care utilisation groups, NT remote Indigenous communities, 2002-2011**

Annual average	Primary care utilisation			95% confidence interval			Reduction	
	Low (n = 6987)	Medium (n = 5926)	High (n = 1271)	Low	Medium	High	Medium	High
Hospitalisations per person								
Diabetes	5 (1421)	1.1 (1892)	1 (772)	4.87-5.11	1.06-1.16	0.89-1.04	78%	80%
IHD	5.9 (612)	2.3 (626)	1.5 (340)	5.69-6.08	2.22-2.47	1.34-1.6	61%	75%
COPD	3.4 (572)	1.3 (657)	1 (322)	3.27-3.58	1.18-1.35	0.92-1.15	62%	71%
Renal Disease	7.7 (1143)	1.2 (1923)	1.1 (826)	7.54-7.87	1.16-1.26	1.06-1.2	84%	86%
Hypertension	4 (1433)	1.1 (1986)	0.9 (832)	3.92-4.13	1.07-1.16	0.88-1.01	73%	78%
PAHs								
Diabetes	2.7	0.6	0.5	2.6-2.78	0.6-0.67	0.48-0.59	76%	80%
IHD	4	1.5	0.9	3.8-4.12	1.43-1.63	0.84-1.05	63%	78%
COPD	2	0.8	0.8	1.93-2.17	0.76-0.9	0.66-0.86	60%	60%
Renal Disease	3.4	0.5	0.6	3.31-3.53	0.5-0.57	0.54-0.65	85%	82%
Hypertension	2.3	0.7	0.5	2.19-2.35	0.64-0.71	0.49-0.6	70%	78%
Deaths (per 100)								
Diabetes	3.23	0.99	0.80	2.92-3.53	0.85-1.14	0.59-1	69%	75%
IHD	4.71	1.76	1.61	4.16-5.26	1.42-2.09	1.17-2.04	63%	66%
COPD	5.08	1.96	2.04	4.48-5.68	1.62-2.31	1.54-2.54	61%	60%
Renal Disease	4.21	1.18	1.06	3.82-4.59	1.02-1.34	0.83-1.29	72%	75%
Hypertension	3.11	1.28	0.97	2.82-3.41	1.12-1.44	0.75-1.19	59%	69%
YLLs								
Diabetes	1.00	0.24	0.20	0.99-1.02	0.23-0.24	0.19-0.21	76%	80%
IHD	1.47	0.46	0.39	1.44-1.5	0.44-0.47	0.37-0.41	69%	73%
COPD	1.32	0.41	0.44	1.29-1.35	0.39-0.42	0.41-0.46	69%	67%
Renal Disease	1.30	0.28	0.25	1.28-1.32	0.27-0.28	0.24-0.27	78%	81%
Hypertension	0.89	0.30	0.23	0.87-0.9	0.29-0.31	0.22-0.24	66%	74%

Notes: n: sample size; COPD: Chronic obstructive pulmonary disease; IHD: Ischemic heart disease; PAH: Potentially avoidable hospitalisations; YLLs: Years of life lost.

Similarly, compared to the low primary care utilisation group, PAH rates in the medium and high primary care groups were 76% and 80% lower, respectively, for diabetes, 63% and 78% for IHD, 70% and 78% for hypertension. For COPD the reduction in the rate of PAH was the same for the both medium and high primary care groups (60%). For renal disease the reduction was greater in the medium compared to the high utilisation group (85% and 82%).

Death rates in the high and medium groups were substantially lower than in the control group for all PCDs. In particular, there were 69% and 75% reductions in death rate for diabetes, and 72% and 75% decreases for renal disease.

Compared to the low primary care group, YLLs were reduced in both the medium and high utilisation groups for all five PCDs. For diabetes, there were 76% and 80% reductions in YLL, respectively, and for renal disease there were 78% and 81% reductions. Overall, reductions in outcomes measured between the low primary care group and the medium group were substantial. Differences between the medium and high primary care groups were marginal. Differences in total hospitalisation rate, PAH and YLL rates between medium and high primary care groups were statistically significant (*p* < 0.05) for all conditions, with the exception of renal disease and COPD. There were no substantial differences in deaths between medium and high utilisation groups (*p* > 0.05).

**Figure 1:**
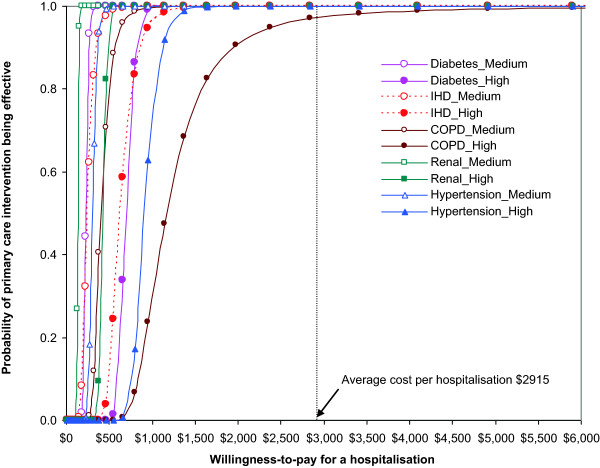
**Cost-effectiveness acceptability curves of primary care intervention for preventable chronic diseases, NT remote Indigenous communities, 2002-2011.**

Figure 1 shows the cost-effectiveness acceptability curves for medium and high level primary care utilisation groups. As the threshold for willingness-to-pay increased, the probability that primary care interventions were more cost-effective also increased, with the medium primary care group more dominant than the high primary care group. Primary care for renal disease was the most cost-effective, followed by diabetes, hypertension and IHD. Primary care for COPD was the least cost-effective among the five PCDs (Figure 1). At the aggregate level, direct hospital savings, defined as the total net benefits for the five PCDs in 2006/7 prices, amounted to $125 million annually, assuming an additive model for the five independent PCDs. Across five PCDs, the cost of saving one hospitalisation was estimated to be in the range of $134-$404 in the medium level of primary care, and $425-$1168 in high level primary care (Table 4). This was far less than the average cost of hospitalisation ($2915) in the NT. It cost $853-$1489 of medium level of primary care and $2581- $4252 of high level of primary care to save one YLL. This compared favourably with the average statistical value of a life year used in this study ($120,000). Investing in either medium or high levels of primary care utilisation for all the PCDs in this study would yield a positive return-on-investment. Estimates of total average savings for investing $1 in a medium level of primary care in remote Indigenous communities could save $11.75 in hospital costs, not including savings in patient transport. Investing an additional $1 in a high level of primary care could save $3.95 in hospital costs. See Table 5.

**Table 4:** **Incremental costs of primary care interventions per hospitalisation averted and year of life saved**

	Medium level of primary care	High level of primary care	Average cost threshold
Per hospitalisation averted	$134 - $404	$425 - $1168	$2915*
Per life year saved	$853 - $1489	$2581 - $4252	$120000^#^

Note: Medium level: 2-11 visits per year; High level: 12 visits per year or more; *Average cost per hospitalisation; ^#^the average statistical value of a life year used.

**Table 5:** **Return on investment ratios of primary care in hospital cost savings by common preventable chronic disease**

	Medium level of primary care	High level of primary care
Diabetes	$12.95	$4.20
IHD	$11.83	$4.61
COPD	$7.21	$2.50
Renal Disease	$21.67	$6.86
Hypertension	$9.72	$3.21
Total average	$11.75	$3.95

Notes: COPD: Chronic obstructive pulmonary disease; IHD: Ischemic heart disease; Medium level: 2-11 visits per year, High level: 12 visits per year or more

In a sensitivity analysis, cut-off points of 0 and 6 visits a year were examined to check if the cut-off points of primary care frequency would have a major impact on the outcomes. The results were not substantially different from those presented.

## Discussion

This large population based observational study using linked data adds to the growing evidence for the cost effectiveness of primary care in remote Indigenous communities [19, 21, 22, 25, 28], where health needs are high, access to primary care is poor and the cost of providing primary care is relatively high, compared to non-remote areas [32]. The results demonstrate that, within the study cohort, medium level primary care use is associated with lower rates of hospitalisation, lower mortality and fewer YLLs. These better health outcomes resulted in significant cost savings for governments through fewer hospitalisations. While investing in primary care does incur costs, they are modest in comparison with the net benefits of providing medium level primary care for the five PCDs. The annual savings of $125 million did not include savings anticipated from chronic diseases other than the five PCDs under study, and benefits anticipated in reductions in YLL.

In a time of fiscal challenge, this evidence is useful to both policy makers and service planners in guiding resource allocation and targeting specific population groups. Improving access to quality primary care is a vital strategy in addressing the burden of PCDs and closing the gap in life expectancy for disadvantaged populations. This can be achieved in several ways. Primary care services must be culturally acceptable to local populations in order to ensure maximum access [15]. Service models such as comprehensive primary health care, provided by remote area nurses, nurse practitioners and Aboriginal health workers, which are broad in scope and address the social determinants of health as well as clinical care, prevention and health promotion are more effective in Indigenous communities [15]. Ensuring quality care through the use of evidence based standard treatment protocols also contributes to better health outcomes [46]. Previous studies have highlighted that for remote areas there are both higher costs of services and funding gaps which result from a lack of doctors who are able to access Medicare [29, 32]. Ensuring the primary care sector receives an equitable, needs-based share of funding in order to redress workforce shortages will contribute to the prevention, early detection and management of PCDs. While the expansion of appropriate, sufficient and high quality services according to need will address much of the primary care service gaps, as demonstrated in this paper, there may be other factors that limit the effectiveness of services in the low utilisation groups. Additional study is needed to identify service models that meet the specific needs for the population at the margins of services. Again in the words of the chair of the Close the Gap steering committee, ‘We should not accept that Indigenous Australians will end up in hospital at twice the rate of other Australians, or suffer significantly higher rates of heart disease, cancer, kidney failure or diabetes. In a wealthy country like Australia, such gross inequality cannot be allowed to continue’.

Limitations: Firstly, we did not have access to data from Aboriginal Community Controlled Health Organisations that also provide primary care in some remote NT communities. However, as the geographical areas were defined by the catchment areas of the clinics, the effects are likely to be minimal. Secondly, we cannot exclude potential selection bias as the high level primary care group might have been relatively sicker than the low utilisation group. Propensity score matching ensured valid comparisons between the three groups and controlled the potential confounders [43]. However, this selection bias is toward the null, thus, if present would result in more conservative outcomes. Thirdly, renal dialysis patients dominated hospital costs. When renal related hospital admissions were excluded, the saving estimates were reduced by 27%. A fourth limitation was that this study considered each PCD independently. Comorbidities and interactions between disease groups were not considered. Fifth, capital costs were not included in this study as they represent long term investments. Sixth, patient migration and change in utilisation through time were beyond the scope of this study. Finally, we did not quantify the social benefits resulting from reductions in YLL nor address other factors that influence PCD including social determinants of health, governance, and sustainable funding models.

## Conclusion

The study demonstrates that increased primary care utilisation is a cost effective way of improving health outcomes for Indigenous people living in remote NT communities. More equitable funding arrangements for the primary care sector would be a well-considered investment in the contribution to closing the gap in Indigenous life expectancy. These findings may have wider applicability in relation to strengthening primary care in the face of high chronic disease prevalence globally.
